# Is serum total bilirubin a predictor of prognosis in arteriosclerotic cardiovascular disease? A meta-analysis

**DOI:** 10.1097/MD.0000000000017544

**Published:** 2019-10-18

**Authors:** Yang Lan, Huan Liu, Jinbo Liu, Hongwei Zhao, Hongyu Wang

**Affiliations:** Vascular Medicine Center, Peking University Shougang Hospital, Beijing, China.

**Keywords:** coronary artery disease, meta-analysis, peripheral arterial disease, serum total bilirubin level, stroke

## Abstract

The protective role of serum total bilirubin, a widely recognized antioxidant, has been approved by numerous updating studies. However, regarding the effect of high serum total bilirubin level (STBL) in arteriosclerotic cardiovascular disease (ASCVD) are conflicting in different sources of data. We, therefore, performed this meta-analysis to evaluate the influence of STBL on risk of ASCVD.

Four databases were used to identify the literature with a date of search of January, 2019. Finally, a total of 20 studies had been adopted. ASCVD was defined as acute coronary syndrome, stable angina, coronary revascularization, atherosclerotic stroke or transient ischemic attack, and peripheral arterial disease (PAD). All relevant data were collected from studies meeting the inclusion criteria.

A total of 20 published studies (323,891 cases) met the inclusion criteria. The meta-analysis revealed that, in studies excluding heterogeneity, STBL was significantly positively related to in-hospital cardiovascular mortality (odds ratio [OR] 2.82, 95% confidence interval [CI] 1.83–4.36, *Z* = 4.69, *P* < .001) and major adverse cardiac events (OR 1.88, 95% CI 1.414–2.491, *Z* = 4.36, *P* < .001), also negatively associated with prognosis of acute myocardial infarction, pooled hazard ratio (HR) = 0.804 (95% CI 0.700–0.923, *Z* = 3.08, *P* = .002). The correlation similarity was also reflected in terms of patients with stroke (HR 0.78, 95% CI 0.70–0.88, *Z* = 4.24, *P* = .003). Combined analysis revealed that lower STBL was significantly associated with PAD, pooled OR = 0.91 (95% CI 0.85–0.98, *Z* = 2.39, *P* = .017). In general analysis, a conclusion can be drawn, that higher STBL was significantly negative correlated with cardiovascular disease, pooled HR = 0.83 (95% CI 0.73–0.94, Z = 3.02, *P* = .003).

Higher STBL significantly improved the prognosis of ASCVD; furthermore, STBL was an important factor in the long-term prognosis of vascular-related disease prevention and can be used as a predictor in vascular-related disease risk prediction.

## 1 Introduction

Cardiovascular disease (CVD) is the leading cause of death not only burden in China also worldwide,^[1]^ accounting for an estimated in every 17.3 million of 54 million total cause of deaths, or responsible of 31.5% all global mortality in 2013.^[2]^ Arteriosclerotic CVD (ASCVD) is defined as coronary artery disease (CAD), stroke, and peripheral arterial disease (PAD), which of these all presumed originated from atherosclerosis.^[3]^ A report from the American Heart Association (AHA) shows, approximately 92.1 million United States adults contracts at least 1 type of CVD and over 15 million people suffer from stroke worldwide, of which 5 million cases of fatality and other 5 million are left with lifetime disability.^[2]^ Consequently, disease burdens of CAD and stroke are increasing rapidly and becoming a serious issue in primary care system.^[2]^ In addition, multiple evidence points to lower extremity arterial ischemia caused by PAD would apparently reduce the quality of life. Therefore, novel and innovative methods are critical in fostering new treatments and improving clinical outcomes in ASCVD.

Bilirubin often has been considered as a nocuousness metabolic waste. However, current understanding redefines it as a potent antioxidant, anti-inflammatory, and neuroprotective molecules. Meanwhile, oxidative stress and inflammation are closely related to the pathogenesis and development of arteriosclerosis.^[4,5]^ Furthermore, arteriosclerosis is the pathologic basis of ASCVD. With the knowledge above, we would come to an assumption that assessing the associations between STBL and risk of ASCVD can be made. Chung et al^[6]^ showed that initial higher STBL was a significant predictor of in-hospital major adverse cardiac events (MACEs) and of cardiovascular death. Huang et al^[7]^ reported that initial STBL was positively correlated with short-term mortality of patients with acute myocardial infarction (AMI). Nevertheless, STBL was negatively correlated with long-term mortality in stable CAD. Zhang et al^[8]^ also revealed that STBL was a protective factor of MACE in patients with CAD. The results of the studies on the associations between STBL and CVD risk were inconsistent. In addition, the protective effect of STBL on ischemic stroke has not been determined. Therefore, we systematically reviewed the observational studies or cohort studies and assessed the association between STBL and the risk of ASCVD.

## 2 Materials and methods

### 2.1 Literature search and studies selection

We systematically researched the studies from the 4 databases including PubMed, Embase, Web of Science, and the Cochrane Library before January, 2019. The search strategies were performed using the following keywords [total bilirubin or bilirubin (MESH)] AND [acute coronary syndrome or stable angina or coronary revascularization, or atherosclerotic stroke or peripheral arterial disease or major adverse cardiovascular events (MESH)]. Two independent authors screened titles and abstracts, and the included articles were reviewed further for full-text reports. Ethical approval was not required because the present study only used the study level data.

The inclusion criteria for the meta-analysis were:

1. Studies assess the relationship between STBL and ASCVD
2. Cohort study or case-control study
3. Studies must provide hazard ratio (HR) or odds ratio (OR) and 95% confidence interval (CI), or give available information calculating HR and 95% CI
4. Studies must be published in English with full-text available

The exclusion criteria for this meta-analysis were:

1. Studies did not report the relationship between STBL and ASCVD
2. Studies were relevant to comments, letters, review articles, and papers lacking statistical information for calculating effect estimates
3. Studies did not involve human, such as animal experiments

### 2.2 Data extraction

Two researchers independently selected the literatures and extracted the data to EXCEL based on the inclusion criteria and exclusion criteria. Disagreements were resolved through discussion or consultation with the 3rd researcher. The data were extracted including: surname of the 1st author, publish year, study design, sample size, gender, mean age at baseline, follow-up periods, events, fully multivariable-adjusted HR/OR and its 95% CI, adjust factors, total bilirubin levels at baseline, diabetes, hypertension, CAD, stroke, and PAD. If the results were analyzed by both univariate and multivariate methods, we chose the latter.

### 2.3 Quality assessment

Assessment of study quality was performed by 2 researchers in our meta-analysis with the standard Newcastle–Ottawa quality assessment scale (NOS), including study population selection, comparability, exposure evaluation, or outcome evaluation. NOS evaluates the quality of the literature by using the semiquantitative principle of the star system, with a maximum of 9 stars.^[9]^ Studies marked 6 or more stars were regarded as high quality articles.

### 2.4 Statistical analysis

All the data were analyzed by STATA software package (Version 12.0; Stata Corp, College Station, TX). HR and 95% CIs had been extracted from each study to assess the relationship between higher STBL and the ASCVD (including AMI, PAD, stroke, stable CAD, and cardiovascular death) and MACE. We assessed the degree of statistical heterogeneity through Cochrane *Q* test and *I*^2^ statistics (ranged from 0% to 100%). By chance statistically significant heterogeneity was observed (*I*^2^ statistic > 50%), we selected a random-effects model. Otherwise, fixed-effects model was chosen. The publication bias of articles by Begg funnel plot was checked. Sensitivity analysis was used to assess the robustness of the results in our analysis. The purpose of sensitivity analysis was to evaluate the effect of a single study on the overall pooled estimates. *P* < .05 was considered statistically significant.

## 3 Results

### 3.1 Selection and characteristics of literature

**Figure 1:**
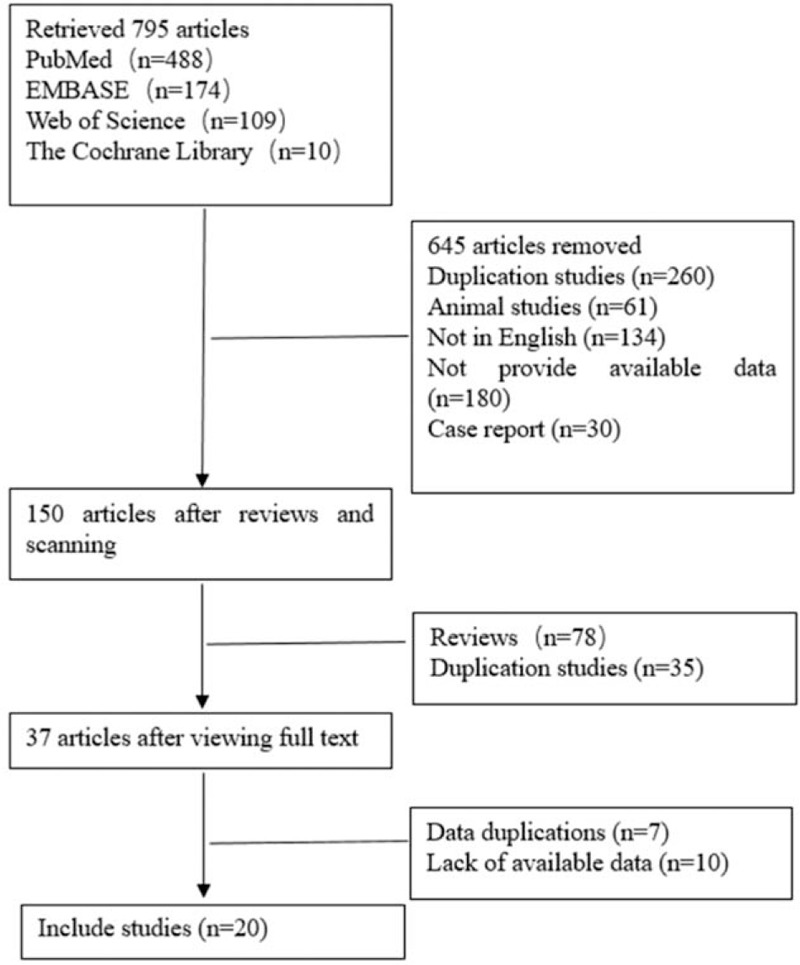
Flow chart showing selection of studies for meta-analysis.

We identified 795 articles from 4 databases regarding the relation of STBL and ASCVD (mainly including stroke, CVD, and PAD) by the above keywords. About 645 studies were excluded 1st by filtering the titles and abstracts for reasons of exclusion study methods (animal experiments, case reports, and reviews) or other languages versatility. After reviewed and assessed full text in detail, 113 literatures were further excluded, reasons of lacking report of HR/OR with 95% CIs, or insufficient data to calculate. To avoid double counting, only 1 article with more available data was selected. Finally, 20 articles involving a total of 323,891 cases were included in the present analysis (Fig. 1). Among the 20 articles, 4^[10–13]^ articles were related to PAD, 3^[14–16]^ were CVD, 7^[11,12,15–19]^ were stroke, and 13^[6–8,11,12,15,16,20–25]^ were CAD. The eligible studies were published ranged from 2008 to 2019, and the sample size ranged from 450 to 113,760. Mean age ranged from 48 to 87 years and male cases ranged from 343 to 41,054. These studies achieved a high-quality score with 5 to 8. Detailed characteristics are summarized in Table 1.

**Table 1:**
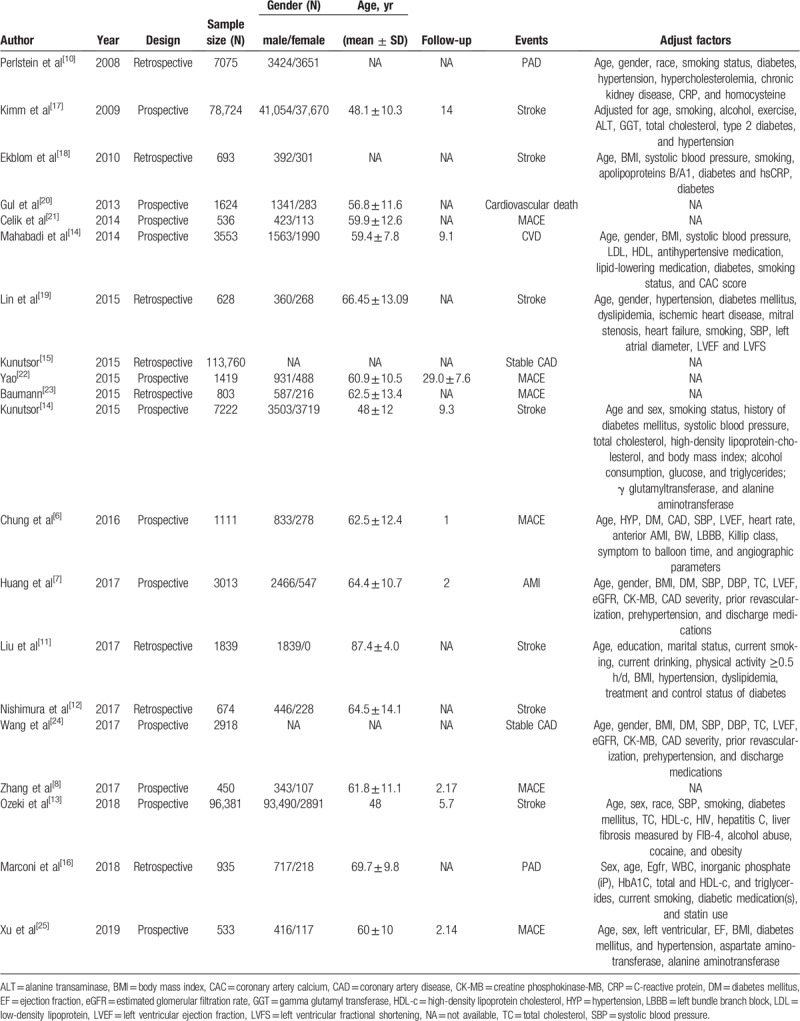
Characteristics of studies included in the meta-analysis.

### 3.2 Relevance between STBL and CAD

**Figure 2:**
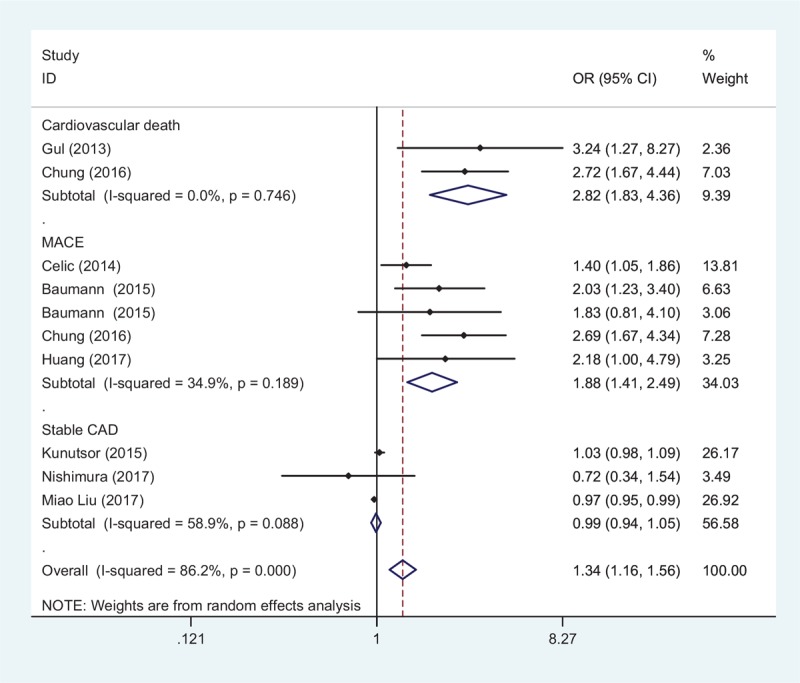
Forest plot of meta-analysis showing higher serum total bilirubin and in-hospital coronary artery disease (CAD; including cardiovascular death, major adverse cardiac events [MACEs], and stable CAD) (pooled with odds ratio [OR]). CI = confidence interval.

**Figure 3:**
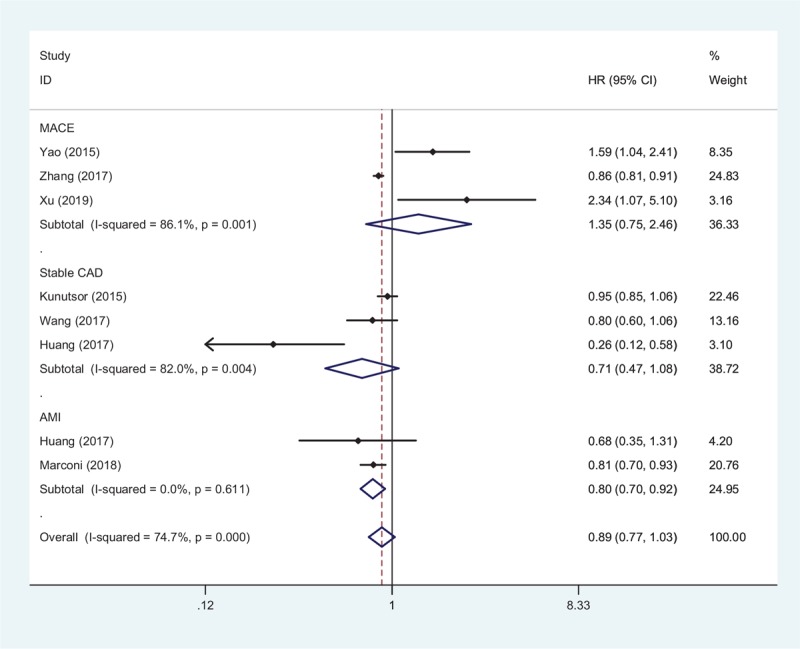
Forest plot of meta-analysis showing higher serum total bilirubin and follow-up coronary artery disease (CAD; including acute myocardial infarction [AMI], major adverse cardiac events [MACE], and stable CAD) (pooled with hazard ratio [HR]). CI = confidence interval.

Thirteen studies provided the data on the relationship between higher STBL and CAD.^[6–8,11,12,15,16,20–25]^ Eight studies reported in-hospital prevalence of CAD^[6,7,11,12,15,20,21,23]^ (2 studies for cardiovascular death, 5 for MACE, and 3 for stable CAD) and 7 studies reported the follow-up prognosis risk of CAD^[7,8,15,16,22,24,25]^ (2 studies for AMI, 3 for MACE, and 3 for stable CAD). All results are shown in Figures 2 and 3. It found that STBL was significantly positively related to in-hospital cardiovascular death (OR 2.82, 95% CI 1.83–4.36, *Z* = 4.69, *P* = .001) and MACE (OR 1.88, 95% CI 1.414–2.491, *Z* = 4.36, *P* = .001). There was a negative significant association of high STBL with prognosis of AMI, with a pooled HR of 0.804 (95% CI 0.700–0.923, *Z* = 3.08, *P* = .002).

### 3.3 Relevance between STBL and stroke

**Figure 4:**
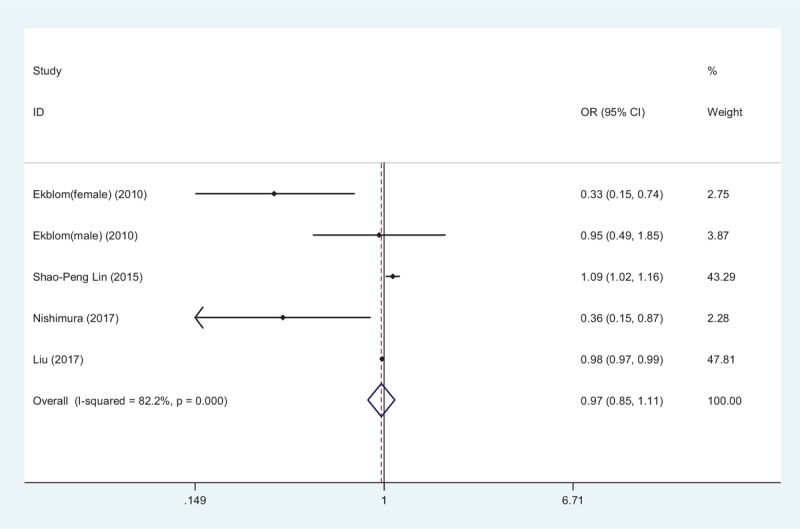
Forest plot of meta-analysis showing higher serum total bilirubin and in-hospital stroke (pooled with odds ratio [OR]). CI = confidence interval.

**Figure 5:**
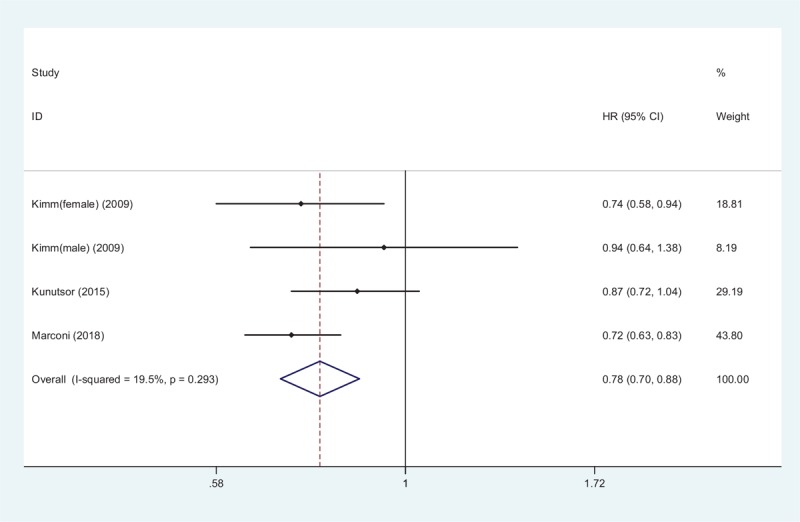
Forest plot of meta-analysis showing higher serum total bilirubin and follow-up stroke (pooled with hazard ratio [HR]). CI = confidence interval.

Seven studies reported the relationship between STBL and stroke.^[11,12,15–19]^ Four studies provided in-hospital prevalence of stroke, and 3 studies provided the long-term prognostic risk of stroke. Figure 4 shows that patients with elevated STBL had 0.97-fold risk of stroke in a random-effect model (OR 0.97, 95% CI 0.85–1.11, *Z* = 0.40, *P* = .69); however, the negative relationship between high STBL and prevalence of stroke was not proved. Nonetheless, in 3 studies with a mean follow-up time of 9.7 years, as shown in Figure 5, it pointed out that higher STBL was a protective maker for stroke prognosis independent of traditional risk factors (HR 0.78, 95% CI 0.70–0.88, *Z* = 4.24, *P* = .003) in a fix-effect model without obvious heterogeneity (*I*^2^ = 19.5%, *P* = .293).

### 3.4 Relevance between STBL and PAD

**Figure 6:**
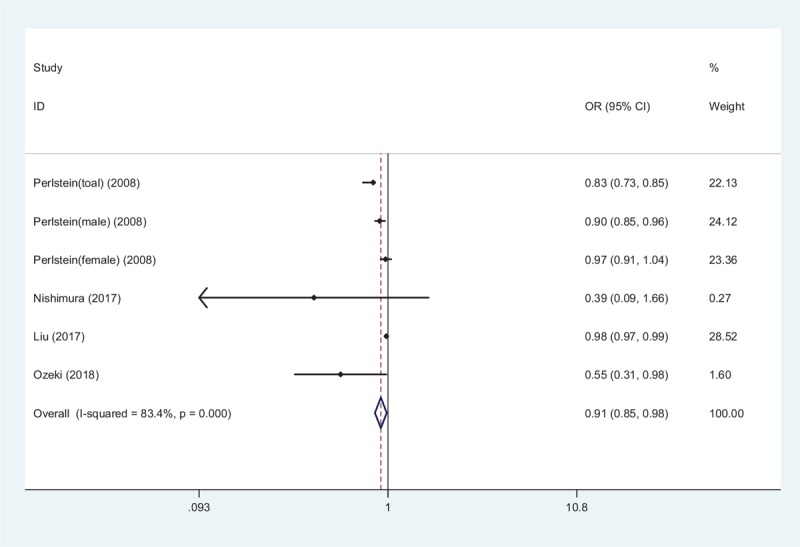
Forest plot of meta-analysis showing higher serum total bilirubin and PAD (pooled with ORs).

Four studies provided a negative correlation between PAD and STBL.^[10–13]^ Combined analysis from all 4 studies revealed that lower STBL was significantly associated with increased prevalence of PAD, with the pooled OR was 0.91 (95% CI 0.85–0.98, *Z* = 2.39, *P* = .017; Fig. 6). There were limited amount of cohort studies on the relationship between PAD and STBL, since that meta-analysis is not performed. However, there was significant heterogeneity among the 4 studies (*I*^2^ = 83.4%, *P* = .001). Sensitivity analysis did not attenuate the combined effect.

### 3.5 Relevance between serum STBL and CVD

**Figure 7:**
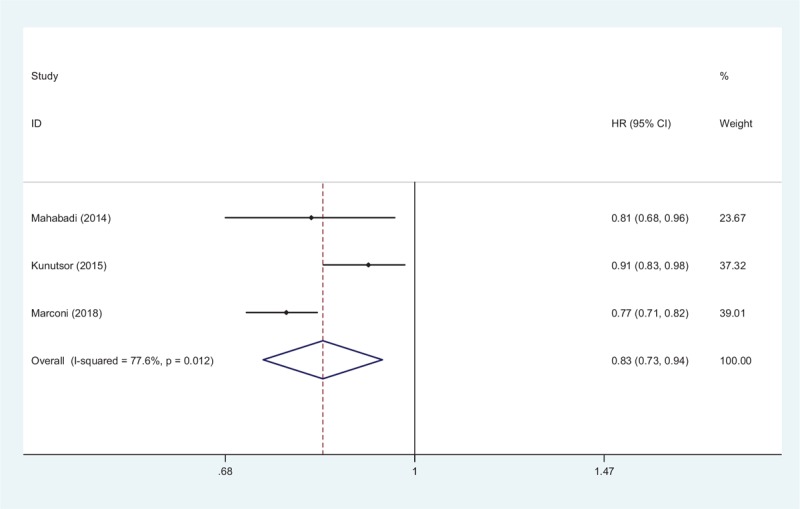
Forest plot of meta-analysis showing higher serum total bilirubin and CVD (pooled with HRs).

Three studies with a mean follow-up time of 8.03 years directly demonstrated the relevance between CVD and STBL.^[14–16]^ Pooled data from all studies indicated that higher STBL was negatively correlated with CVD, with a combined HR value was 0.83 (95% CI 0.73–0.94, *Z* = 3.02, *P* = .003; Fig. 7). Sensitive analysis showed that the existence of the study of Kunutsor^[15]^ mainly accounted for heterogeneity of the meta-analysis (*I*^2^ = 77.6%, *P* = .012). After removal of this study, the heterogeneity confounders disappeared completely (*I*^2^ = 0.0%, *P* = 0.595), results revealed that higher STBL was a protective factor for the prognosis of CVD (HR 0.78, 95% CI 0.73–0.98, *P* = .001).

### 3.6 Publication bias

**Figure 8:**
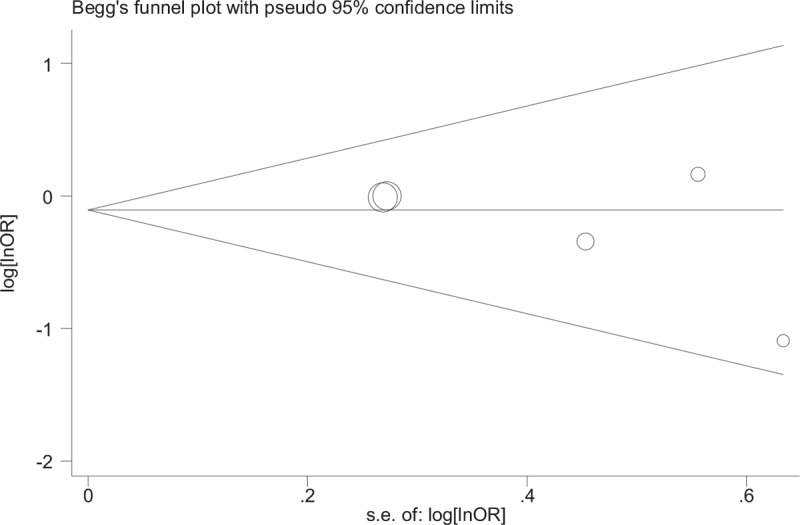
Begg funnel plot for assessing publication bias in in-hospital coronary artery disease.

**Figure 9:**
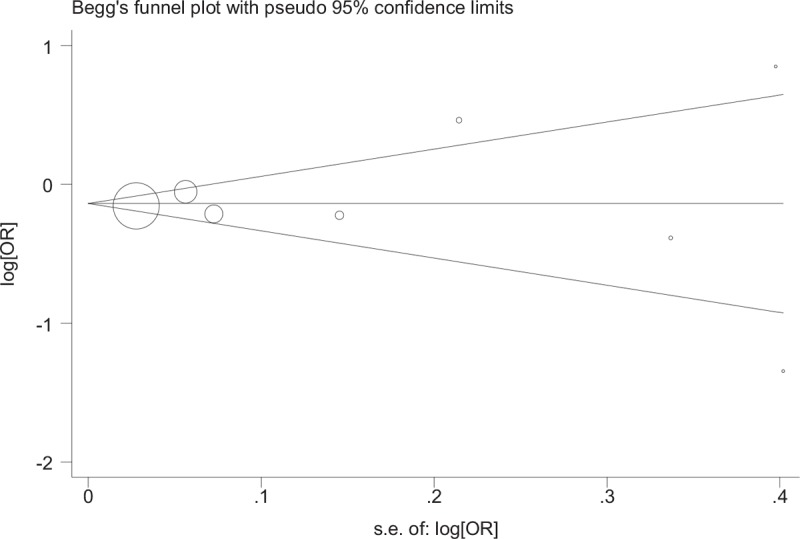
Begg funnel plot for assessing publication bias in follow-up coronary artery disease.

**Figure 10:**
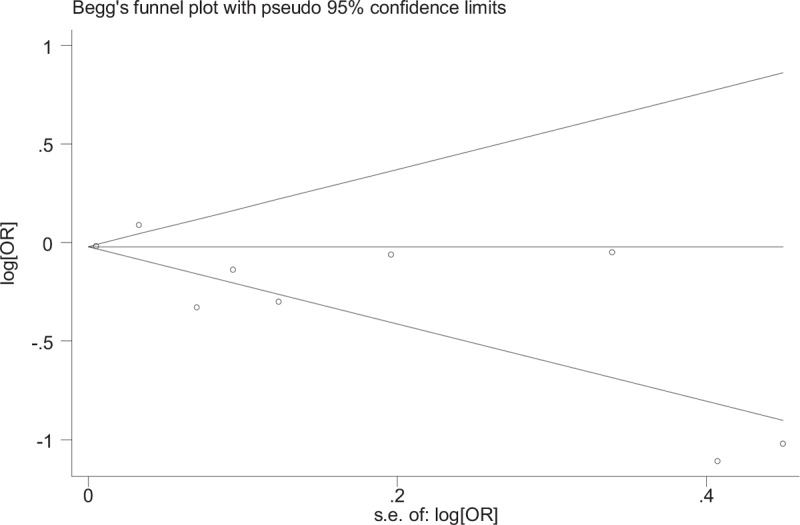
Begg funnel plot for assessing publication bias in stroke.

The results of our meta-analysis did not discover evidence of publication bias nor funnel plot asymmetry in studies on CAD and stroke (Figs. 8–10). Begg test to determine the publication bias was not preformed due to minor studies on PAD were included, also results of subgroup analysis may be unreliable.

## 4 Discussion

Meta-analysis has been wildly recognized as an effective method to direct clinical practice or research by systematically combining and reviewing previous published researches to arrive at conclusions about the body of research. The results from previous studies on the relationship between the higher STBL and the prognosis of ASCVD have been inconsistent. In the present meta-analysis, 20 published articles, namely, 13 studies on CAD, 7 studies on stroke, 4 studies on PAD, and 3 studies on CVD, involving a total of 323,891 patients were included. Few prospective cohort studies on PAD and STBL, and the pooled HR for PAD and STBL were not analyzed. The results indicated that higher STBL was an independent protective factor for ASCVD and negatively associated with the prognosis of stroke, AMI, and PAD, but positively associated with in-hospital cardiovascular death and MACE.

Bilirubin is an endogenous antioxidant, which resist for oxidative modification of low-density lipoprotein cholesterol, participate in scavenging oxygen-free radicals, and increase heme oxygenase activity and the ability of serum cholesterol to dissolve.^[26,27]^ Elevated STBL is significantly positively associated with in-hospital cardiovascular death and MACE, and a recent meta-analysis strongly supported for our findings.^[28]^ Heme oxygenase-1 (HO-1) is the rate-limiting enzyme of bilirubin and mediates the conversion of heme to bilirubin. The activity of stress-inducing HO-1 enzyme in patients with AMI is independently associated with coronary blood flow damage.^[21]^ Initial serum bilirubin levels (STBLs) may reflect of the severity of acute myocardial damage. The level of bilirubin increased transiently in the acute phase of AMI and returned to the normal instantly, and the prognostic value of the initial STBL was reduced.^[29]^ Our analysis revealed that elevated STBL was associated with the decreasing of risk in long-term death, which was consistent with previous reported results. Studies also showed the decreased risk of CAD in individuals with high STBL.^[7,11]^

Based on our analysis, we determined the inverse association between bilirubin and follow-up stroke by combining the published studies. The results in the cohort studies supported for the potential role of bilirubin in the prognosis of stroke. Unfortunately, we did not determine the protective effect of higher STBL on in-hospital stroke, and several studies failed to show a causal relationship between genetically elevated bilirubin and stroke.^[30]^ Otherwise, patients in acute stroke showed higher STBL, results of low hemodynamic status.^[30]^ High STBL was linked to low prevalence of PAD. STBL was also known as heat shock protein, which could be induced in ischemic conditions to mitigate ischemic tissue injury.^[13]^ Patients with relatively lower STBL had high risk of tissue hypoxia related to the HO-1 gene variation.^[31]^

The possible mechanisms of underlying the relationship between high STBL and better prognosis of ASCVD were as followed: bilirubin could effectively block the generation of cellular reactive oxygen species induced by the cross-linking of endothelial vascular cell adhesion molecule 1 and intercellular adhesion molecule 1, and further prevent the formation of atherosclerotic plaque^[32]^; STBL respectively resists for myeloperoxidase-induced protein or lipid oxidation and scavenge hypochlorous acid to prevent the formation of atherosclerosis^[33]^; and higher STBL also resist for an anti-inflammatory effect on atherosclerotic process. STBL was inversely correlated with inflammatory markers including C-reactive protein, neutrophil to lymphocyte ratio, and red cell distribution in patient with coronary atherosclerosis.^[34]^ In addition, bilirubin partly inhibits the induction of complement through anti-apoptosis,^[35]^ regulates the activity of various T lymphocytes,^[36]^ and reduces the production of proinflammatory cytokines.^[37]^

There are still limitations in our meta-analysis to be discussed. There are limited prospective studies on PAD risk and STBL, we particularly combined the OR of high STBL to PAD and did not comprehensively assess the risk for increased STBL and PAD prognosis, and revealing the potential protective effects lacked enough evidence by the retrospective analysis. Thus, potential publication bias may exist, although there was no evidence obtained from our statistical tests. Another weakness of this analysis is that partial of the results was inconsistent with previous studies, and large-sample, long-term prospective cohort studies were required to validate our results. Finally, the follow-up periods and the cutoff value of STBL in baseline were incongruent.

## 5 Conclusion

In conclusion, higher STBL could decrease the future risk of ASCVD and provide a better prognosis of ASCVD, while the increased prevalence of in-hospital CAD (including in-hospital MACE or cardiovascular death) and in-hospital stroke may exhibit higher STBL levels.

## Author contributions

**Conceptualization:** Yang Lan, Jinbo Liu.

**Data curation:** Yang Lan, Huan Liu, Hongwei Zhao.

**Formal analysis:** Yang Lan, Huan Liu, Hongwei Zhao.

**Funding acquisition:** Jinbo Liu.

**Investigation:** Hongwei Zhao.

**Methodology:** Jinbo Liu.

**Project administration:** Jinbo Liu.

**Resources:** Jinbo Liu.

**Software:** Yang Lan, Huan Liu, Jinbo Liu.

**Supervision:** Huan Liu, Jinbo Liu.

**Validation:** Hongwei Zhao.

**Writing – original draft:** Yang Lan.

**Writing – review & editing:** Jinbo Liu, Hongyu Wang.
